# A prospective multiple case study of the impact of emerging scientific evidence on established colorectal cancer screening programs: a study protocol

**DOI:** 10.1186/1748-5908-7-51

**Published:** 2012-06-01

**Authors:** Hannah Geddie, Mark J Dobrow, Jeffrey S Hoch, Linda Rabeneck

**Affiliations:** 1 Cancer Services & Policy Research Unit, Cancer Care Ontario, Toronto, Ontario, Canada; 2 Health Policy Management & Evaluation, University of Toronto, Toronto, Ontario, Canada; 3 Pharmacoeconomics Research Unit, Cancer Care Ontario, Toronto, Ontario, Canada; 4 Keenan Research Centre, Li Ka Shing Knowledge Institute, St. Michael’s Hospital, Toronto, Ontario, Canada; 5 Prevention and Cancer Control, Cancer Care Ontario, Toronto, Ontario, Canada

**Keywords:** Evidence-based health policy, Policy decision making, Population-based colorectal cancer screening

## Abstract

**Background:**

Health-policy decision making is a complex and dynamic process, for which strong evidentiary support is required. This includes scientifically produced research, as well as information that relates to the context in which the decision takes place. Unlike scientific evidence, this “contextual evidence” is highly variable and often includes information that is not scientifically produced, drawn from sources such as political judgement, program management experience and knowledge, or public values. As the policy decision-making process is variable and difficult to evaluate, it is often unclear how this heterogeneous evidence is identified and incorporated into “evidence-based policy” decisions. Population-based colorectal cancer screening poses an ideal context in which to examine these issues. In Canada, colorectal cancer screening programs have been established in several provinces over the past five years, based on the fecal occult blood test (FOBT) or the fecal immunochemical test. However, as these programs develop, new scientific evidence for screening continues to emerge. Recently published randomized controlled trials suggest that the use of flexible sigmoidoscopy for population-based screening may pose a greater reduction in mortality than the FOBT. This raises the important question of how policy makers will address this evidence, given that screening programs are being established or are already in place. This study will examine these issues prospectively and will focus on how policy makers monitor emerging scientific evidence and how both scientific and contextual evidence are identified and applied for decisions about health system improvement.

**Methods:**

This study will employ a prospective multiple case study design, involving participants from Ontario, Alberta, Manitoba, Nova Scotia, and Quebec. In each province, data will be collected via document analysis and key informant interviews. Documents will include policy briefs, reports, meeting minutes, media releases, and correspondence. Interviews will be conducted in person with senior administrative leaders, government officials, screening experts, and high-level cancer system stakeholders.

**Discussion:**

The proposed study comprises the third and final phase of an Emerging Team grant to address the challenges of health-policy decision making and colorectal cancer screening decisions in Canada. This study will contribute a unique prospective look at how policy makers address new, emerging scientific evidence in several different policy environments and at different stages of program planning and implementation. Findings will provide important insight into the various approaches that are or should be used to monitor emerging evidence, the relative importance of scientific versus contextual evidence for decision making, and the tools and processes that may be important to support challenging health-policy decisions.

## Background

Health-policy decision making poses many challenges. There is an unprecedented proliferation of scientific information available to policy makers, and with it, increasing demand to ensure that decisions are based on the highest-quality up-to-date evidence [1]. Similar challenges in clinical practice have resulted in strong emphasis being placed on evidence-based medicine, a linear process of decision making in which scientific evidence is applied to resolve identified “knowledge gaps” [2]. Increasingly, the concept of evidence-based decision making is applied to health policy and is frequently cited as a political or tactical tool to suggest an approach that results in higher-quality policy decisions [1]. However, while the application of evidence-based medicine to resolve clinical questions is widely accepted, “evidence-based health policy” (EBHP) is far less clear [1-3].

Unlike clinical decisions, policy decisions are inherently context-specific [1,2] and require a diverse array of evidence and stakeholder involvement at various stages of the decision-making process [1,2]. Those who participate in health-policy decision making may range from senior government and administrative officials, trained methodologists and clinicians, program managers, or community stakeholders [1]. These actors are “anchored within institutional settings” and often have diverse perspectives and interpretations of relevant evidence [1,4]. In a report by Lomas *et al.*, the heterogeneity of evidence used for health-policy decision making was characterized as “scientific context-free,” “scientific context-sensitive,” and “colloquial” [5]. In this typology, an important distinction is drawn between evidence generated via scientific methods that describes what works under ideal circumstances, scientific evidence that refers to a specific context, and colloquial evidence that broadly implies “anything that establishes a fact or gives reason for believing in something” (*i.e.*, stakeholder opinion and values) [5]. To address complex policy decisions, policy makers must often “identify,” “interpret,” and “apply” each of these forms of evidence in terms of their relevance to the decision at hand [5,6]. However, while scientific and colloquial evidence pertaining to the local context (hereafter referred to under the broad heading of “contextual evidence”) play a crucial role in decision making, this role is often unclear. Unlike non-context-specific evidence published in scientific journals, contextual evidence is often harder to define and to obtain. It does not fit properly within the evidence-based framework and thus there is a lack of clarity as to how it impacts decision making.

Finally, unlike clinical decisions, multifactorial policy decisions are not outlined in clear linear stages [1,2]. Instead, they comprise a series of objectives/foci, including efficacy/effectiveness (will it work), appropriateness/feasibility (should we do it), and implementability (how do we do it here) [6]. Increasingly there appears to be more focus on questions related to implementation [6], which corresponds to the difficulties policy makers face in allocating resources and planning complex programs. For challenging implementation-related questions, if contextual evidence is ill defined or unavailable, this may result in program-management approaches dominating evidence-based approaches when decisions must be made [7]. Ultimately, due to the challenges of evaluating the policy decision-making process and the lack of clarity in how evidence is used, it is often very difficult to identify what is an EBHP decision or how it was reached.

### Situating the study

Colorectal cancer (CRC) screening presents an ideal context to examine the challenges of health-policy decision making. The second most common cause of cancer deaths in Canada [8], CRC has a well-known path of disease progression and vastly improved chances of survival with early diagnosis [9]. However, while there is strong evidence to support population-based screening, there are fewer definitive answers as to the characteristics of the screening program that should be in place and how to implement them. There are a variety of modalities to screen for CRC, each of which poses various advantages and disadvantages for population-based programs. In Canada, screening decisions have been made in several provinces based on the implementation of the fecal occult blood test (FOBT) or fecal immunochemical test (FIT), yet questions still remain as to the optimal design of these programs. Meanwhile, new evidence continues to emerge, bringing renewed perspective to the CRC screening policy debate. In a recent UK-based trial by Atkin *et al.*[10], the use of once-only flexible sigmoidoscopy (FS) for population-based screening demonstrated a significant reduction in CRC-related mortality, suggesting even greater population-wide benefit than the biennial screening with FOBT currently in place in most provincial programs [11]. This trial is one of four major randomized controlled trials, including the SCORE [12], NORCCAP [13], and PLCO [14] trials, that together provide high-quality evidence of the utility of FS for population-based screening. As evidence mounts, policy makers will need to decide on the relevance of these findings for the screening programs currently in place.

In examining this subject, the proposed study builds on previous formative research on the use of evidence to inform complex cancer-screening policy decisions. From 2002–2006, Dobrow *et al.* retrospectively examined how four expert groups in Ontario made screening policy recommendations for four types of cancer screening (colorectal, breast, cervical, and prostate) [6]. With the insight gained from this work, a second retrospective study was conducted to more closely examine screening decisions for a single disease site (CRC) across different jurisdictions. This study found that even with the same evidence and guidance from advisory bodies, significant differences among the provinces were observed in terms of what constituted the ultimate decision to establish screening, the key questions asked, and how evidence was used. Preparation of a manuscript of this work is currently in progress.

This study comprises the final phase of this work and will examine prospectively how emerging scientific evidence on FS is addressed by policy makers in five provinces. This study reflects an important progression in our work, revisiting the same five provinces and providing a new and innovative perspective in an area in which relatively little is known—the impact of emerging scientific evidence when policy decisions have already been made. This issue is timely and relevant in Canada, as screening programs continue to be developed and implemented, and the Canadian Partnership Against Cancer has convened an anticipatory science panel to examine new evidence on FS [11]. This issue is also of relevance internationally, given the wide variability in programs that have been implemented and ongoing interest in evaluating how these decisions are and continue to be made [7,15]. This study’s research team is in a unique position to consider these issues and to provide findings that are insightful to population-based screening programs in Canada and elsewhere.

### Hypotheses/assumptions

In developing the methodology and rationale for this study, we incorporate some key assumptions. Primarily, we assume policy makers use a variety of different approaches to monitor and identify emerging scientific evidence and that these approaches will vary based on jurisdiction and stakeholder group. We also make the assumption that there are limited formal tools and guidance to support policy makers in integrating emerging scientific evidence with relevant contextual information.

We intend to approach policy makers with the question of whether to incorporate FS into population-based programs and hypothetically how this decision should be made. Consequently, we anticipate that policy makers will not provide a definitive yes/no response but will discuss gaps in contextual information that must be filled, particularly pertaining to program implementation, and context-specific factors that would determine how this issue is addressed.

### Research questions

Based on the above, this study will address the following research questions:

1.) How do policy makers monitor and become aware of emerging scientific evidence?

2.) How does new scientific evidence impact established screening programs/policies?

3.) How do policy makers identify/interpret contextual evidence?

4.) How does contextual evidence impact established screening programs/policies?

5.) How do policy makers integrate scientific with contextual evidence to address decisions about health system improvement?

## Methods/Design

This study will employ a prospective, multiple case study design involving participants from Ontario, Manitoba, Alberta, Nova Scotia, and Quebec. Primary methods for data collection will include document analysis and semi-structured interviews in each province.

Documents relevant to each province’s consideration of evidence for CRC-screening decisions will be collected. A systematic search strategy will be used to collect these data, beginning with documents available in the public domain, followed by requests to key informants and organizations for further material. Documents will include policy recommendations/reports/briefs, planning documents, meeting minutes, media releases, and correspondence.

Interviews will be conducted with five to eight policy makers and cancer system stakeholders in each province, including administrative leaders, government officials, screening experts, and other high-level stakeholders from ministries of health and cancer agencies. Purposive sampling will be used to identify these informants, with snowball sampling used subsequently to further identify individuals involved in CRC-screening decisions for the province. All potential interviewees will be sent an introductory package explaining the project and describing procedures for confidentiality and informed consent. The research team will then contact the individual by phone and arrange a convenient interview time. Interviews will be conducted in person and will last approximately 60 minutes each. Any additional interviews will be conducted by telephone. All participants will be asked to sign consent forms prior to the interview. Interviews will be audio-recorded and transcribed verbatim where permission is granted. Data will be analyzed using NVivo (QSR International, Cambridge, MA, USA) qualitative analysis software. For this project, ethical approval has been sought and obtained from the University of Toronto’s Research Ethics Board.

This study will be completed in accordance with the timeline of our Canadian Institutes of Health Services Research Emerging Team Grant. It is anticipated that the data collection phase of this work, including document collection and key informant interviews, will be completed by September 2012, with transcription and coding of the interview data to be completed by the end of the calendar year. Following this, the analysis and write up of findings will take place and will be completed by March 31, 2013.

## Discussion

As scientific evidence continues to emerge, posing new opportunities for health system improvement, it is important to examine how policy makers will respond. Primarily, the findings of this study will provide insight into how emerging scientific evidence is monitored, and how this varies across stakeholder groups and jurisdictions. If key informants are already aware of emerging evidence on FS and engaging in steps to address this evidence, study findings will allow for comparison of what methods and processes are currently being used. Conversely, if key informants are unaware or unable to discuss emerging scientific evidence, study findings will provide valuable insight into what methods/processes should be in place for this (if any), and how to better support policy makers in this important area.

Furthermore, this study will provide important insight into the relative importance of scientific versus contextual evidence for decisions about health system change. Although scientific evidence is disseminated on a continual basis, evidence appears to be less clear for program and implementation-related questions that pose significant challenges for policy makers. For this, the multiple case study design is ideal and will allow for comparison of ways in which contextual evidence is identified and applied in five unique jurisdictions at various stages of program planning and implementation.

Finally, in evaluating health-policy decision making, research is often reactive. Instead of examining retrospectively how decisions have been made, this study will provide unique insight into policy decision making from a hypothetical and prospective sense. We hope to contribute positively to the EBHP literature and to help support and advance the use of evidence in making challenging policy decisions that profoundly impact our healthcare system.

## **Competing interests**

The authors declare that they have no competing interests.

## **Authors’ contributions**

All authors contributed to the original concept and methodology of this study. The initial draft of this manuscript was completed by HG. All authors reviewed the manuscript, provided critical comments and suggestions for revision, and ultimately approved the final completed work. All authors read and approved the final manuscript.
